# Alcohol as a Dressing to Wounds

**Published:** 1869-06

**Authors:** W. F. McNutt


ALCOHOL AS A DRESSING TO WOUNDS. By W. F. McNutt, M. D.—The advantages claimed for alcohol as a dressing to surgical and traumatic wounds are, that in recent wounds it coagulates the soluble albumen on the surface of the wound, corrugates the tissues, and contracts the small vessels, thereby preventing the accumulation of blood or serum between flaps or the edges of wounds, which would necessarily prevent primary union.

Applied as a dressing to granulating wounds, it acts as a local stimulant, prevents largely the formation of pus, lessens the chances of the patient's having pyæmia, is an excellent disinfectant, and possesses the advantage of being a stimulant to the genetal system.

